# Interventional Radiology in Acute Cholecystitis: A Review of Contemporary Percutaneous Strategies and Emerging Techniques

**DOI:** 10.3390/jcm15135106

**Published:** 2026-06-30

**Authors:** Dimitrios Giannis, Panagiota Gianni

**Affiliations:** 1 Department of Surgery, Flushing Hospital Medical Center, MediSys Health Network, Queens, NY 11355, USA; 2 Department of Internal Medicine III, Hematology, Oncology, Palliative Medicine, Rheumatology and Infectious Diseases, University Hospital Ulm, 89081 Ulm, Germany; panagiota.gianni@uniklinik-ulm.de

**Keywords:** acute cholecystitis, interventional radiology, acute care surgery, frailty, critical care, percutaneous cholecystostomy

## Abstract

**Background/Objectives**: Acute cholecystitis is a common surgical emergency associated with significant morbidity in elderly, frail, and critically ill poor surgical candidates. Early laparoscopic cholecystectomy remains the standard of care for low-risk patients, but interventional radiology (IR) modalities have been increasingly used in high-risk patients for gallbladder decompression, source control, and/or definitive non-operative treatment. **Methods**: A narrative review of the literature was performed to investigate current percutaneous IR options in acute cholecystitis. Evidence from international guidelines, randomized trials, systematic reviews, meta-analyses, and experimental novel techniques were reviewed. The patient selection approaches, timing of intervention, efficacy, complications, and risk of recurrence were summarized. **Results**: Percutaneous cholecystostomy remains the most commonly performed IR procedure for acute cholecystitis, offering decompression and source control in patients unfit for surgery. Percutaneous gallstone extraction and gallbladder chemical ablation, or cryoablation, have been used to reduce recurrence and long-term catheter dependence with promising results, but are still limited by complications and insufficient evidence. The variability in practice patterns and the absence of standardized treatment algorithms contribute to mixed results, ranging from long-term/definitive symptom control to the prolonged dependence on indwelling catheters and readmissions for catheter-related complications. **Conclusions**: IR plays an important role in the management of high-risk patients with acute cholecystitis. The careful selection of patients based on disease severity, physiologic reserve, frailty, and patient-centered goals is frequently limited by institutional resources. A structured clinical decision framework to guide IR-based interventions in acute cholecystitis is of the utmost importance to achieve optimal outcomes. Future studies should focus on standardized algorithms, patient-centered outcomes, recurrence, tube-free survival, and quality of life.

## 1. Introduction

Acute cholecystitis is most commonly caused by the obstruction of the cystic duct due to a gallstone (calculous cholecystitis) [1,2], and, less frequently, due to cholestasis in critically ill patients (acalculous cholecystitis) [3]. Acute cholecystitis is responsible for approximately 3–10% of hospital admissions in patients presenting with acute abdominal pain, with an estimated annual incidence of 200,000–300,000 cases in the United States [4].

Laparoscopic cholecystectomy (LC) is the gold standard in patients that are optimized for surgery. The outcomes after LC are better in younger, healthy surgical candidates, but morbidity and mortality increase significantly in older, frail patients with comorbidities [5,6]. Emergency surgery in high-risk patients is associated with a higher rate of postoperative complications, prolonged intensive care unit (ICU) stay, loss of functional independence, and higher mortality [7,8,9].

Early source control is a central principle in the management of acute cholecystitis. Timely intervention can halt the inflammatory process, prevent gallbladder gangrene or perforation, and reduce the risk of systemic complications such as septic shock or multi-organ failure [4]. Interventional Radiology (IR) procedures (percutaneous cholecystostomy (PCT), chemical ablation, cryoablation, and gallstone extraction) have been used with increasing frequency and promising results in patients that are not surgical candidates.

The aim of this narrative review is to summarize the established and emerging IR techniques for the management of acute cholecystitis within an evidence-based decision-making framework.

## 2. Methods

This narrative review was conducted to summarize contemporary percutaneous interventional radiology approaches for the management of acute cholecystitis. A literature search was performed using PubMed/MEDLINE, Embase, and Google Scholar for studies published through January 2026. Search terms included combinations of “acute cholecystitis”, “acalculous cholecystitis”, “percutaneous cholecystostomy”, “gallbladder drainage”, “interventional radiology”, “gallstone extraction”, “ablation”, “cryoablation”, “frailty”, and “high-risk surgical patients”. English-language publications were prioritized. Evidence from international guidelines, randomized controlled trials, observational studies, systematic reviews, meta-analyses, and technical reports was reviewed. Priority was given to studies evaluating patient selection, clinical outcomes, complications, and recurrence. As a narrative review, formal study selection criteria, risk-of-bias assessment, and quantitative synthesis (meta-analysis) were not performed.

For the purposes of this review, “high-risk” patients refer to individuals with increased perioperative morbidity and mortality based on factors such as advanced age, severe comorbidity burden, American Society of Anesthesiologists (ASA) class III–IV, Tokyo Guidelines 2018 (TG18) Grade II–III acute cholecystitis, organ dysfunction, or ICU admission. “Frailty” refers to diminished physiologic reserve and vulnerability to adverse outcomes. “Poor surgical candidates” are patients in whom operative intervention is considered to carry excessive risk because of severe cardiopulmonary disease, advanced frailty, multiple comorbidities (Charlson Comorbidity Index (CCI) ≥ 5), organ failure, or limited functional status. “Limited life expectancy” refers to patients with advanced chronic illness, end-stage organ disease, advanced malignancy, or severe functional dependence in whom long-term survival is expected to be substantially reduced. Technical success is defined as the successful completion of the intended IR procedure as planned, with correct placement of catheter or completion of the targeted intervention, confirmed intra-procedurally or immediately post-procedure. Clinical success is defined as the resolution of acute cholecystitis following the procedure, without need for additional urgent intervention, as reflected by clinical, laboratory, and/or imaging improvement.

## 3. Percutaneous Cholecystostomy Tube

### 3.1. Background

Percutaneous cholecystostomy tube (PCT) has been the standard approach to temporize poor surgical candidates [5,10] (Table 1 and Table 2). PCT was first described in the early 1980s as a minimally invasive technique for high-risk patients, as a temporizing measure before interval LC and as a definitive treatment in elderly or critically ill patients with multiple comorbidities and short life-expectancy [11].

PCT is performed transhepatic or transperitoneal and its success depends on complications, catheter stability, and subsequent management. Transhepatic access offers a more secure catheter tract and decreased rates of bile leakage, while transperitoneal access may be preferred in patients with coagulation abnormalities or altered hepatic anatomy. Current evidence suggests comparable overall success rates between the two approaches, but higher dislodgement rates with the transperitoneal route [7,8].

The TG18, which provides an international framework for the diagnosis, severity grading, and treatment in acute cholecystitis, recommends PCT as a safe and effective bridging therapy, especially for high-risk patients with Grade II/III acute cholecystitis or patients that require stabilization before definitive surgery [4]. More recently, the CHOCOLATE trial and a meta-analysis summarizing evidence from 27 studies suggest that early LC may provide superior long-term outcomes, including mortality and readmission rates, compared with PCT [6,18].

Currently, there is a significant gap between guideline recommendations and real-world practice, since a considerable proportion of patients never undergo drain removal or interval surgery, often due to comorbidities, frailty, or loss to follow-up [11,19,20,21]. This discrepancy highlights the ongoing challenges between guideline-directed management and consistent post-procedure care in high-risk and resource-limited settings.

### 3.2. Timing and Mortality

The current evidence shows that delayed PCT (defined as PCT placement performed more than 3–4 days from hospital admission) is associated with significantly higher mortality [9]. Khasawneh et al. reported an approximately two-fold higher 30-day mortality when PCT was delayed beyond 4 days, even after adjusting for comorbidities [22]. Further, ICU admission at the time of PCT placement is considered a marker of disease severity and has been associated with worse outcomes [23]. In this setting, prolonged inflammation may result in a more technically challenging drainage procedure, a longer catheter dwell time, a higher rate of tube occlusion or dislodgement, and an increased risk of recurrent cholecystitis while awaiting further intervention [24]. These findings reinforce the necessity of establishing institutional protocols that prioritize PCT placement within the first 72 h of presentation for high-risk patients with acute cholecystitis, particularly those demonstrating rapid clinical deterioration or escalating inflammatory markers.

### 3.3. Catheter Management

The optimal dwell time for PCT catheters remains unclear [9]. According to the TG18, the catheter should remain in place for at least three weeks to promote tract maturation and the resolution of inflammation [4]. The concept of “dual timing” dictates the minimum safe dwell time to avoid complications associated with premature removal and the maximum time beyond which the risk of infection and patient discomfort increase substantially [25]. Lin et al. suggest the upper safe limit to be around 6–8 weeks, as extended dwell times result in increased rates of infection, granulation tissue formation, and difficult tube removal [26].

Catheter care is important to prevent complications and maintain patency. The drain should be secured without tension, the insertion site should be maintained clean and dry and inspected daily for signs of infection or bile leakage. Routine catheter irrigation with 5–10 mL of sterile saline solution, once or twice daily, is recommended to prevent occlusion [27]. Patients and caregivers should be educated on proper irrigation technique, dressing changes, local wound care, and early signs of catheter dysfunction, such as new onset pain, bile leakage, or decreased output. Detailed printed instructions and follow-up phone calls may improve adherence and reduce visits to the emergency department for tube-related issues. Real-world challenges include patient and family language barriers, poor compliance with outpatient follow-up visits, delays in scheduling cholangiograms, and barriers between IR, medical, and surgical providers.

Based on these findings, a standardized removal protocol, that includes a three- to six-week dwell time, daily catheter irrigation, and cholangiogram to confirm the patency of the cystic duct prior to PCT removal, may reduce complications and facilitate timely catheter discontinuation.

### 3.4. Interval Cholecystectomy After PCT

Definitive cholecystectomy after stabilization with PCT is a critical step in the long-term management of acute cholecystitis. Interval LC eliminates the source of gallstones and the risk of recurrent cholecystitis, biliary colic, cholangitis, and gallstone pancreatitis [28]. LC following PCT has been associated with better long-term outcomes compared with drainage alone [29]. The need for interval LC after PCT varies between calculous and acalculous cholecystitis. In calculous cholecystitis, recurrence rates can be as high as 40% without surgery. Lower recurrence rates (9.2–23.5%) have been reported when a cholangiogram is performed to confirm the patency of the cystic duct before removing the PCT [30,31,32]. In contrast, acalculous cholecystitis, commonly seen in critically ill patients, is a result of ischemia, stasis, or systemic inflammation rather than mechanical obstruction. Once acalculous cholecystitis resolves, the risk of recurrence is lower and any benefit of LC is substantially smaller [21,33].

The most common barriers in poor surgical patients after PCT include medical comorbidities, patient preference, and loss to follow-up. Financial reasons and socioeconomic factors may delay follow-up and access to surgery [21,34,35,36]. A structured reassessment protocol should include a plan for follow-up and LC as an outpatient procedure, the reassessment of PCT and surgical candidacy at predefined intervals, and the integration of patient navigation services to minimize loss to follow-up.

According to the TG18 [4] and the 2020 update from the World Society of Emergency Surgery [5], there is no universally defined timeframe for interval cholecystectomy after PCT. These guidelines advocate for a bridge-to-surgery strategy, recommending that definitive cholecystectomy be performed once the patient’s overall condition has stabilized and any comorbidities have been appropriately optimized. This approach favors clinical judgment over defined intervals. Recent evidence showed that early interval LC, performed within the first two to four weeks after drainage, may be associated with increased technical difficulty and complication rates, while delayed LC beyond eight weeks may predispose patients to recurrent biliary events. In summary, the current evidence suggests that performing LC at approximately four to eight weeks after PCT provides a balanced and safe approach for most patients that are medically optimized [9,27].

Despite the benefits of PCT, recent evidence points toward a gradual shift in the management of severe acute cholecystitis. Based on improved laparoscopic techniques, perioperative optimization, and newer antibiotics, recent studies have demonstrated the feasibility and safety of early LC in carefully selected high-risk populations [6,37]. Further experience and evidence from larger prospective trials with long-term follow-up are still required before early LC can be considered a safe option in high-risk candidates [6,38,39].

### 3.5. Limitations and Future Directions

PCT has been increasingly used over the past few decades mostly due to an aging population with multiple comorbidities [20]. Recurrent cholecystitis after PCT has been reported in up to 46%. Complications of PCT include sepsis (5%), major bleeding (1.4%), hollow viscus perforation (1.4%), catheter dislodgement (7%), bile leak (2.8%), clogged catheter (7%), and minor bleeding (1.2%) [40,41,42,43,44].

Overall mortality and complications after PCT are associated with disease severity, comorbidities, and timing of intervention. A higher number of comorbidities has been associated with higher mortality after PCT [33]. Early intervention protocols, integrating risk assessment scores such as the CCI, and prioritizing timely drainage in patients with a worsening clinical status may improve outcomes. Lastly, the available evidence is mostly observational and subject to residual confounding. Increased mortality observed with delayed drainage may reflect a greater disease severity, delayed diagnosis, or underlying patient factors rather than a direct causal effect of timing alone.

## 4. Ablation Techniques

### 4.1. Background

A major determinant of the resolution of chronic cholecystitis is the complete destruction of the mucosal layer of the gallbladder. Residual mucosa produces mucin and increases the risk of mucocele formation and recurrent cholecystitis [45]. Further, it has been hypothesized that the metaplasia of the muscularis layer results in mucosal regeneration and an increased risk of recurrence [46]. Ablative therapies cause irreversible injury and fibrosis across the full thickness of the gallbladder wall. Chemical (sclerosing agents) and cryoablative modalities induce protein denaturation, membrane rupture, microvascular thrombosis, and ischemic necrosis [47,48]. Cryoablation, in particular, causes intracellular ice crystal formation and osmotic cell lysis during freeze–thaw cycles [49].

### 4.2. Chemical Gallbladder Ablation

Chemical gallbladder ablation using sclerosing agents such as ethanol or acetic acid has been investigated in high-risk patients with acute or recurrent cholecystitis. The proposed mechanism involves the chemical destruction of the gallbladder mucosa, leading to transmural inflammation, fibrosis, contraction, and functional obliteration. Early observational series with cholecystostomy placement followed by the instillation of 95% ethanol, showed sustained symptom relief in 85.3% of high-risk patients [50]. Later, Lee et al. showed the technical feasibility and short-term symptom control via chemical ablation with pure alcohol through an established PCT [47]. In a prospective single-center study by Atar et al., cystic duct embolization was added to chemical ablation via PCT in high-risk elderly patients. Technical success was achieved in all cases, without immediate procedure-related complications at a median follow-up of 11 months [15]. Recently, Le Tat et al. conducted a retrospective study evaluating percutaneous ethanol ablation with or without cystic duct embolization in high-risk elderly patients with acute cholecystitis. The procedure was technically feasible in 20 patients (9 underwent cystic duct embolization) and was performed without major procedural complications and without long-term PCT dependence. Follow-up imaging demonstrated gallbladder atrophy, particularly in patients that underwent cystic duct embolization [16].

Despite promising findings, the application of chemical ablation has remained limited due to the inconsistent outcomes and the absence of standardized protocols regarding the agent concentration, dwell time, and number of sessions. Chemical ablation appears less predictable, both in the extent of the gallbladder wall damage and in long-term efficacy. The distribution of sclerosants within the gallbladder varies and depends on cystic duct patency, gallbladder morphology, and the presence of stones or debris, which may shield portions of the mucosa from exposure [16,51,52]. Early animal studies evaluating multiple chemical sclerosants showed that a uniform mucosal destruction was difficult to achieve with ethanol alone or other agents, and portions of the mucosa could remain viable depending on the agent type and degree of exposure [53]. Transmural gallbladder wall sclerosis may not be achieved, because these agents do not penetrate the Rokitanksy–Aschoff sinuses consistently, which are thought to be responsible for mucosal regeneration. Residual mucosa and a patent cystic duct increase the risk of recurrent cholecystitis [46]. In addition, procedure-related morbidity has limited the utilization of chemical sclerosis [47,54,55]. Procedure-related complications such as gallbladder mucocele, hydrops, abscess formation, and perforation have been described [47,50,53,54].

Beyond technical considerations, an ongoing debate is whether chemical ablation via PCT offers any benefit or if it is overtreatment, particularly in older frail patients. In patients with a limited life expectancy, the primary goal is to achieve source control in a timely manner rather than the prevention of long-term recurrence. In this population, PCT already offers adequate short-term palliation with lower procedural risk [56,57]. The incremental benefit of gallbladder ablation is therefore uncertain, especially when weighed against the increased pain, complications, and prolonged recovery [4,57]. Chemical ablation may have a role in select survivors with recurrent cholecystitis, persistent tube dependence, and reasonable functional reserve, but it cannot be justified as a routine treatment based on the current evidence.

### 4.3. Gallbladder Cryoablation

Cryoablation is a method of tissue destruction through the application of freezing low-temperature followed by rapid thawing that damages the cell membranes and induces cell death/necrosis [49,58]. Cryoablation is a well-established intervention for a wide variety of clinical settings, such as malignancy or neurolysis and the devices used in cryoablation have evolved since its inception, making it less invasive over the years [59] and safe when applied to pathology adjacent to major hepatic blood vessels, bowel, and the common bile duct [60,61]. In addition, the real-time intraprocedural imaging of the ablation margins facilitates accurate lesion coverage and limited non-target injury to adjacent tissues [62]. As a result of these advancements, cryoablation has been recently utilized as an alternative definitive treatment for cholecystitis in poor surgical candidates [1,48].

Cryoablation is performed with the placement of a PCT, hydrodissection, the image-guided placement of probes, surrounding tissue displacement with saline, and alternating freeze/thaw cycles [48]. The placement of a PCT facilitates the decompression of the gallbladder and decreases the size of the ablation zone required [1,17]. Hydrodissection is performed to physically and thermally isolate adjacent tissues such as the duodenum, the colon, and the common bile duct. Probes are placed under CT and/or ultrasound guidance into and close to the gallbladder and cystic duct, and ablation is performed through 10-min freeze cycles until a 5 mm margin of ablated tissue is visualized on imaging [45].

Cryoablation causes direct cold-induced cellular damage (cellular shrinkage or crystal formation during freezing cycles, and cellular edema during thawing), induces cellular death, and affects the cellular microenvironment (damage of vascular endothelial cells, thrombosis/ischemia, release of cytokines, tissue edema, and influx of inflammatory cells) [49,63]. Cryoablation causes transmural necrosis with margins that include the mucosa and muscularis. Cryoablation has the advantage of inducing comparable cellular damage and deeper injury without affecting the adjacent parenchyma compared to heat-based ablation [64]. The ability to monitor the size of the ice ball during cryoablation further reduces the risk of damage to surrounding tissues [45].

The clinical success of cryoablation varies between 90–100% [1,17]. The first study of cryoablation in cholecystitis was conducted by McGregor et al. in 2019 [65]. The procedure was performed with three probes and two freeze cycles, followed by the removal of the PCT and the patient remained symptom-free at 3 months. This initial success was followed by a retrospective cohort of six patients published by the same researchers in 2020 showing promising results at a mean follow-up of 9 months. Patients were free of abdominal pain, and serial imaging at 3-month intervals up to 1 year showed the involution of the gallbladder in five out of six patients. Two adverse events included post-operative infection and severe hemorrhage causing acute kidney injury and the need for hemodialysis [66]. Based on these findings, the same research group conducted a prospective study in ten patients with chronic cholecystitis that were previously treated with PCT placement [17]. At a mean follow-up of approximately 1.5 years with CT or MRI imaging, the gallbladder was involuted in nine out of ten patients. The two adverse events included post-operative infection and one patient death after anaphylactic shock, both occurring in patients with gallstones larger than 2 cm, a history of pseudomonas infection, and an ice ball size greater than 150 cm^3^. These findings emphasize the need for the careful selection of patients that undergo cryoablation, and the need for future studies to identify populations that may benefit the most, considering the comorbidities, the amount and size of gallstones, and the optimal size of the ice ball that would achieve the best outcome and maximum safety. More recently, Abraham et al. reported a dual-center retrospective cohort of 12 patients treated with gallbladder cryoablation combined with gallstone extraction for calculous cholecystitis and cryoablation alone for acalculous disease [1]. Technical success was achieved in 91.7%, with all successfully treated patients being symptom-free at 1 month and at a median follow-up of approximately 18 months. Serial imaging showed progressive gallbladder involution without mucosal enhancement. There were no immediate procedure-related complications and only two minor delayed adverse events were reported (scrotal edema and transient biliary obstruction). These findings indicate that stone extraction before cryoablation may decrease the risk of infection, decrease the required ablation volume, and improve the procedural safety in this high-risk population. Despite these promising results, patient selection for cryoablation remains a critical issue. In older patients or those with a limited life expectancy, the incremental benefit of cryoablation over gallstone extraction alone is uncertain. At present, there are no standardized criteria and decisions are individualized based on surgical risk, comorbidity burden, anticipated survival, and likelihood of recurrent disease. Further studies are needed to define which subgroups derive a durable benefit from gallbladder devitalization, and to identify the thresholds of age, comorbidity, and recurrence risk at which cryoablation provides a clinical advantage over less invasive interventions.

## 5. Percutaneous Gallstone Extraction

Percutaneous gallstone extraction is performed under imaging guidance (ultrasound, fluoroscopy, and cholangioscopy) and involves percutaneous access through an established cholecystostomy tract and techniques like basket retrieval, laser/ultrasonic lithotripsy (stone crushing), and saline irrigation.

Early experience with percutaneous cholecystolithotomy demonstrated promising technical success and acceptable safety in high-risk patients. In a retrospective series of 75 patients (ASA classification III–IV), Stirrat et al. reported a technical success (complete gallstone removal) rate of 90.7% and low readmission rates (4% at 30 days and 8% at 3 months). Procedure-related complications (10.4%) during a mean follow-up of 2.8 ± 3.7 years were minor (Clavien–Dindo I–II), including postoperative choledocholithiasis in 8%, while 6% developed recurrent cholelithiasis requiring cholecystectomy or PCT replacement. Most patients remained free of gallstone-related complications (77.3%) during follow-up [12].

Similarly, a recent multi-institutional study of 30 consecutive patients that underwent percutaneous gallstone extraction through cholangioscopy reported a 93% technical success, including patients with large stones up to 4 cm in diameter. Most patients achieved complete stone removal in a single session, had low complication rates (6.7%), and remained asymptomatic at the 3-month follow-up [13].

A systematic review by Latif et al. evaluated the safety and efficacy of percutaneous cholecystolithotomy and included 12 studies comprising 435 high-risk patients. The pooled analysis demonstrated an overall technical success rate of 91%, at the cost of an overall complication rate of 28%, including both minor and major adverse events, while the procedure-related mortality was 0.7% [67]. A more recent systematic review and meta-analysis of 13 studies by Nadeem et al. evaluated percutaneous cholecystolithotomy/lithotripsy for acute calculous cholecystitis in non-surgical candidates and demonstrated a high technical success rate (97%) with low rates of stone recurrence (10%) and recurrent cholecystitis (1%). The reported complications included retained stones (3%), duct perforation (6%), catheter displacement (5%), bleeding (4%), and bile leakage (5%) [14]. Based on these pooled analyses, percutaneous gallstone extraction appears to be effective in high-risk patients but is associated with a relatively high rate of adverse events.

Recent advances in cholangioscopy and lithotripsy devices and techniques have further facilitated gallstone extraction as an option. Data from the National Percutaneous Cholangioscopy Registry demonstrated high clinical success rates for both percutaneous electrohydraulic lithotripsy and percutaneous laser lithotripsy, achieving symptom resolution in 92–98% of cases. Notably, adverse event rates were low (4.3% and 3.3%, respectively), and biliary drainage tubes were successfully removed in 66–76% during follow-up [68].

These findings support the idea that percutaneous gallstone extraction may function as a bridge towards definitive management in carefully selected patients, particularly those with recurrent episodes but a reasonable functional reserve. Despite its conceptual appeal, percutaneous gallstone extraction remains underutilized due to the technical complexity and limited operator experience. The procedure is painful and requires a mature tract, and experience with fluoroscopy or cholangioscopy and retrieval devices, all of which may not be available outside high-volume centers [69]. The number and size of the stones and complex gallbladder anatomy further affect the technical success. These barriers have prevented widespread adoption, particularly in resource-limited settings.

Another important limitation is the risk of recurrence, which persists even after a successful gallstone extraction. Gallbladder dysmotility, residual microlithiasis, and ongoing lithogenic bile composition may predispose patients to stone formation and recurrent inflammation [70].

The absence of standardized protocols represents a major barrier to broader clinical integration. There is no consensus regarding patient selection, the optimal timing after cholecystostomy, the choice of extraction devices, or the role of lithotripsy and chemical dissolution. Outcomes are heterogeneous, without a standardized follow-up or patient-centered endpoints, such as quality of life or tube-free survival [14]. As a result, percutaneous gallstone extraction remains a niche intervention rather than a routine component of acute cholecystitis management. Future prospective studies are needed to identify patients that would benefit the most from gallstone extraction.

## 6. Clinical Decision Framework for IR in Acute Cholecystitis and Ethical Boundaries

Frailty has emerged as one of the strongest independent predictors of mortality, complications, and morbidity in patients with acute cholecystitis [71,72]. In this population, IR techniques provide rapid source control while avoiding the physiologic stress of emergent surgery. However, the availability of minimally invasive approaches also introduces ethical complexity, as technical success does not necessarily translate into a meaningful clinical benefit for patients with advanced frailty, poor baseline function, or limited life expectancy.

Critically ill or severely frail patients that undergo IR interventions depend on external drains, are at risk of recurrent infections or reinterventions, and participate in complicated goals-of-care discussions. For example, a PCT may effectively control sepsis. yet commit a patient to months of tube care, repeat exchanges, cholangiograms, and recurrent admissions without a clear path to definitive treatment. In such scenarios, there is no clear distinction between life-prolonging therapy and experimental care in a patient with limited life expectancy, particularly when further procedures (stone extraction or gallbladder ablation) are performed without clear evidence.

These considerations are especially relevant in very elderly patients with acute cholecystitis, in whom the natural history of the disease may be overshadowed by competing risks of death or functional decline from non-biliary causes. While IR techniques reduce the peri-procedural risk compared with surgery, they are still associated with a high morbidity. Ethical decision-making in this setting requires clinicians to distinguish interventions that meaningfully alter the disease trajectory (tube-free survival) from those that merely extend treatment duration without improving survival, quality of life, or independence.

Accordingly, a structured reassessment after the initial IR intervention is essential. Clear, predefined endpoints—such as the resolution of sepsis, candidacy for tube removal, or transition to comfort-focused care—should be established early in the course of treatment. This approach ensures that IR serves as a tool for goal-concordant care rather than an automatic default in patients for whom the burdens of ongoing intervention may outweigh the benefits.

**Figure 1:**
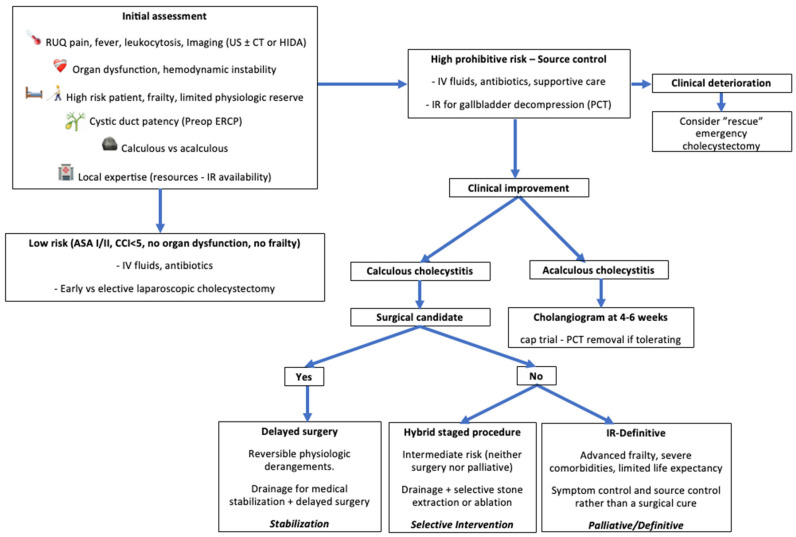
Decision framework for the use of interventional radiology in acute cholecystitis.

Based on the current evidence, we propose a three-domain clinical decision framework that integrates patient risk, disease biology, and technical feasibility to guide the appropriate use of surgery and interventional radiology (Figure 1). Patient risk encompasses not only chronological age, but also frailty, physiologic reserve, organ dysfunction, and anticipated recovery potential. Disease biology includes the severity of cholecystitis, the presence of complications such as empyema or gangrene, cystic duct patency, and the likelihood of recurrence. Technical feasibility reflects local expertise, resource availability, and the relative risks of surgical versus percutaneous approaches. Within this framework, the following treatment pathways should be considered:

- The surgery-first approach remains appropriate for physiologically robust patients with mild to moderate acute cholecystitis, in whom early laparoscopic cholecystectomy offers definitive treatment and minimizes recurrence;
- An IR-first, bridge-to-surgery strategy is best suited for patients with reversible physiologic derangements, allowing time for stabilization before delayed cholecystectomy;
- Hybrid/staged approaches, including drainage followed by selective gallstone extraction or ablation techniques, may benefit patients with intermediate risk who are neither ideal operative candidates nor clearly palliative;
- An IR-definitive (lifelong PCT, ablation) or palliative strategy is most appropriate for patients with advanced frailty, severe comorbidities, or limited life expectancy, in whom the goal is symptom control rather than definitive cure.

Importantly, management within this framework should consider the goals of care and ongoing reassessment.

## 7. Conclusions

IR has fundamentally reshaped the management of acute cholecystitis and enabled timely source control in high-risk patients. IR techniques complement surgical management with treatment options across a spectrum of physiologic reserves. However, the use of IR procedures should be considered along with goals-of-care discussions and ethical boundaries, especially in older, frail patients.

A structured clinical decision framework is essential to ensure that IR enhances, rather than replaces, sound surgical judgment and goal-concordant care. By integrating patient-centered outcomes, disease biology, and technical feasibility, clinicians can better align intervention intensity with the anticipated benefit, ensuring that advances in IR translate into better outcomes for patients with acute cholecystitis.

## Figures and Tables

**Table 1:** Summary of percutaneous interventional radiology techniques for cholecystitis.

Technique	Primary Indication	Mechanism/Goal	Clinical Success	Major Advantages	Major Limitations/ Complications	Typical Role
Percutaneous cholecystostomy tube (PCT)	High-risk surgical candidates with acute calculous or acalculous cholecystitis	Gallbladder decompression and source control	>90%	Sepsis control in poor candidates	Catheter dislodgement/occlusion, bile leak, infection, recurrent cholecystitis, prolonged tube dependence	Bridge to surgery or definitive/palliative therapy
Percutaneous gallstone extraction	Patients unfit for surgery with persistent stones or recurrent calculous cholecystitis	Removal of gallstones through mature cholecystostomy tract using baskets, lithotripsy, or cholangioscopy	77.3–98%	Gallbladder preservation, tube removal possible, low recurrence of cholecystitis in selected cohorts	Technically challenging, painful, recurrence of stones, tract maturation and expertise needed	Definitive nonoperative management in select patients
Chemical gallbladder ablation	Recurrent cholecystitis or persistent tube dependence in nonsurgical candidates	Ethanol/acetic acid-induced mucosal destruction and fibrosis	85.3–100%	Potential definitive therapy without surgery	Inconsistent ablation, mucocele, abscess, perforation	Selective adjunctive therapy
Gallbladder cryoablation	Chronic/recurrent cholecystitis in poor operative candidates after drainage	Freeze–thaw cycles causing transmural necrosis and gallbladder fibrosis	90–100%	Image-guided monitoring, deeper tissue penetration, preservation of adjacent structures	Limited evidence, bleeding/infection risk, uncertain long-term benefit in frail patients	Emerging definitive therapy in carefully selected patients

**Table 2:** Key studies and outcomes of percutaneous interventional radiology approaches in acute cholecystitis.

Study	Intervention	Study Design	Population	Main Findings
Loozen et al. (CHOCOLATE trial), 2018 [6]	Early laparoscopic cholecystectomy vs. PCT	Multicenter randomized trial	High-risk acute cholecystitis patients	Early laparoscopic cholecystectomy associated with fewer complications and recurrence compared with PCT
Stirrat et al., 2021 [12]	Percutaneous gallstone extraction	Retrospective cohort	75 high-risk patients	Technical success 90.7%; free from recurrent gallstone complications 77.3%
Smirniotopoulos et al., 2024 [13]	Cholangioscopy-assisted gallstone extraction	Multi-institutional retrospective study	30 patients	Technical success 93.3%; low complication rates (6.7%); durable symptom control at 3 months (100%)
Nadeem et al., 2025 [14]	Percutaneous cholecystolithotomy/lithotripsy	Systematic review/meta-analysis	13 studies	Technical success 97%; recurrent cholecystitis 1%
Atar et al., 2020 [15]	Chemical gallbladder ablation with cystic duct embolization	Prospective single-center study	High-risk elderly patients	Technical success 100%; no immediate complications at 11 months
Le Tat et al., 2025 [16]	Ethanol gallbladder ablation with/without cystic duct embolization	Retrospective study	Elderly inoperable patients	Feasible with gallbladder atrophy on follow-up imaging
McGregor et al., 2025 [17]	Gallbladder cryoablation	Prospective trial	10 patients	Gallbladder involution in 90% at 18 months follow up
Abraham et al., 2025 [1]	Cryoablation with/without stone extraction	Dual-center retrospective series	12 patients	Technical success 91.7%; sustained symptom resolution at median 18 months
Fanciulli et al., 2025 [18]	Cholecystectomy vs. PCT + delayed cholecystectomy vs. PCT only	Systematic review/meta-analysis	27 studies	Cholecystectomy was associated with lower mortality and readmissions compared to drainage and delayed surgery or drainage only

## Data Availability

The data presented in this review are publicly available and can be found in the references of the study.
